# First detection of *Paenibacillus larvae* the causative agent of American Foulbrood in a Ugandan honeybee colony

**DOI:** 10.1186/s40064-016-2767-3

**Published:** 2016-07-15

**Authors:** Moses Chemurot, Marleen Brunain, Anne M. Akol, Tine Descamps, Dirk C. de Graaf

**Affiliations:** Laboratory of Molecular Entomology and Bee Pathology, Ghent University, Krijgslaan 281 S2, 9000 Ghent, Belgium; Department of Zoology, Entomology and Fisheries Sciences, College of Natural Sciences, Makerere University, P.O. Box 7062, Kampala, Uganda

**Keywords:** *Apis mellifera*, Bacterial pathogens, East Africa, Honeybee diseases, Prevalence

## Abstract

*Paenibacillus larvae* is a highly contagious and often lethal widely distributed pathogen of honeybees, *Apis mellifera* but has not been reported in eastern Africa to date. We investigated the presence of *P. larvae* in the eastern and western highland agro-ecological zones of Uganda by collecting brood and honey samples from 67 honeybee colonies in two sampling occasions and cultivated them for *P. larvae*. Also, 8 honeys imported and locally retailed in Uganda were sampled and cultivated for *P. larvae*. Our aim was to establish the presence and distribution of *P. larvae* in honeybee populations in the two highland agro-ecological zones of Uganda and to determine if honeys that were locally retailed contained this lethal pathogen. One honeybee colony without clinical symptoms for *P. larvae* in an apiary located in a protected area of the western highlands of Uganda was found positive for *P. larvae*. The strain of this *P. larvae* was genotyped and found to be ERIC I. In order to compare its virulence with *P. larvae* reference strains, in vitro infection experiments were conducted with carniolan honeybee larvae from the research laboratory at Ghent University, Belgium. The results show that the virulence of the *P. larvae* strain found in Uganda was at least equally high. The epidemiological implication of the presence of *P. larvae* in a protected area is discussed.

## Background

*Paenibacillus larvae* is a spore forming gram-positive bacterial pathogen of the European honeybees, *Apis mellifera*. *P. larvae* affects honeybee brood causing American Foulbrood (AFB) which is a highly contagious and often lethal disease in managed honeybee colonies. The disease poses a significant threat to the health of honeybee colonies and to the beekeeping industry because it causes considerable losses to beekeepers (Genersch et al. 2005; Genersch 2010b). Honeybee larvae get infected when they are fed by nurse bees on feed contaminated with spores of *P. larvae*. Young larvae (<36 h after hatching) are most susceptible to infection (Genersch 2010a). A dose of about 10 spores or fewer is sufficient to successfully infect and kill honeybee larva (Woodrow 1942). Typical clinical symptoms of AFB are the brown, viscous larval remains forming a ropy thread when drawn out with a matchstick (de Graaf et al. 2006). The decaying brood desiccates into hard scales, tightly adhering to the walls of the cells, consisting of millions of bacterial spores which are the infectious stage of the pathogen (Genersch et al. 2006).

AFB is spread both horizontally and vertically (Fries et al. 2006; Lindstrom et al. 2008). However, the most predominant route of spread is via the horizontal routes by both humans and bees. Horizontal transmission of AFB occurs when humans move contaminated honey or beekeeping equipment (Genersch 2010a). In addition, drifting of adult bees between colonies and robbing behavior of foragers can lead to horizontal spread of AFB (Lindstrom et al. 2008).

*Paenibacillus larvae* has a worldwide distribution (Matheson 1993; Genersch 2010a; Human et al. 2011; Morrissey et al. 2015). However, with the exception of Eritrea, Gambia, Guinea-Bissau, Senegal, South Africa (Matheson 1996; Hansen et al. 2003; Ellis and Munn 2005; de Graaf et al. 2006; Human et al. 2011), Tunisia (Matheson 1993; Hussein 2001; Fries and Raina 2003; Hamdi et al. 2013), Algeria (Adjlane et al. 2014), Libya and Morocco (Hussein 2001; Pirk et al. 2015) there is still doubt whether *P. larvae* is present in most parts of Africa since no confirmations have been made.

Beekeeping is an important activity in many rural areas of Uganda where it is carried out mainly using traditional beehives and beekeeping practices (UEPB 2005; Chemurot 2011). Beekeepers in Uganda majorly target honey production (UEPB 2005), although beekeeping provides several other benefits to people and the environment: production of propolis, beeswax, bee venom, pollen and pollination service (Jacobs et al. 2006; Genersch 2010b). In doing so, honeybees contribute to food security and biodiversity conservation. However, honeybees are threatened by numerous pathogens like viruses, bacteria, fungi and parasites which can attack them. The most recent honeybee parasite reported in Uganda, is *Varroa destructor* (Chemurot et al. 2016). In order to develop the beekeeping sector, it is essential to design effective honeybee pest and disease management plans. This requires accurate and adequate information on the distribution of honeybee pathogens in the country.

As part of a bigger project investigating the distribution of honeybee pathogens in selected agro-ecological zones of Uganda, we collected brood and honey samples from honeybee colonies in the eastern and western highland agro-ecological zones (AEZ) of Uganda. We also collected 8 honey samples from imported honeys that are locally retailed in Uganda and cultivated them for *P. larvae*. The aim of this study was to establish the presence and distribution of *P. larvae* in honeybee populations in two highland agro-ecological zones of Uganda and to determine if honeys that were locally retailed contained this lethal pathogen. Here we present data showing the presence of *P. larvae* in one honeybee colony without any clinical symptoms and discuss the epidemiological implication of the findings.

## Methods

### Study area

**Fig. 1:**
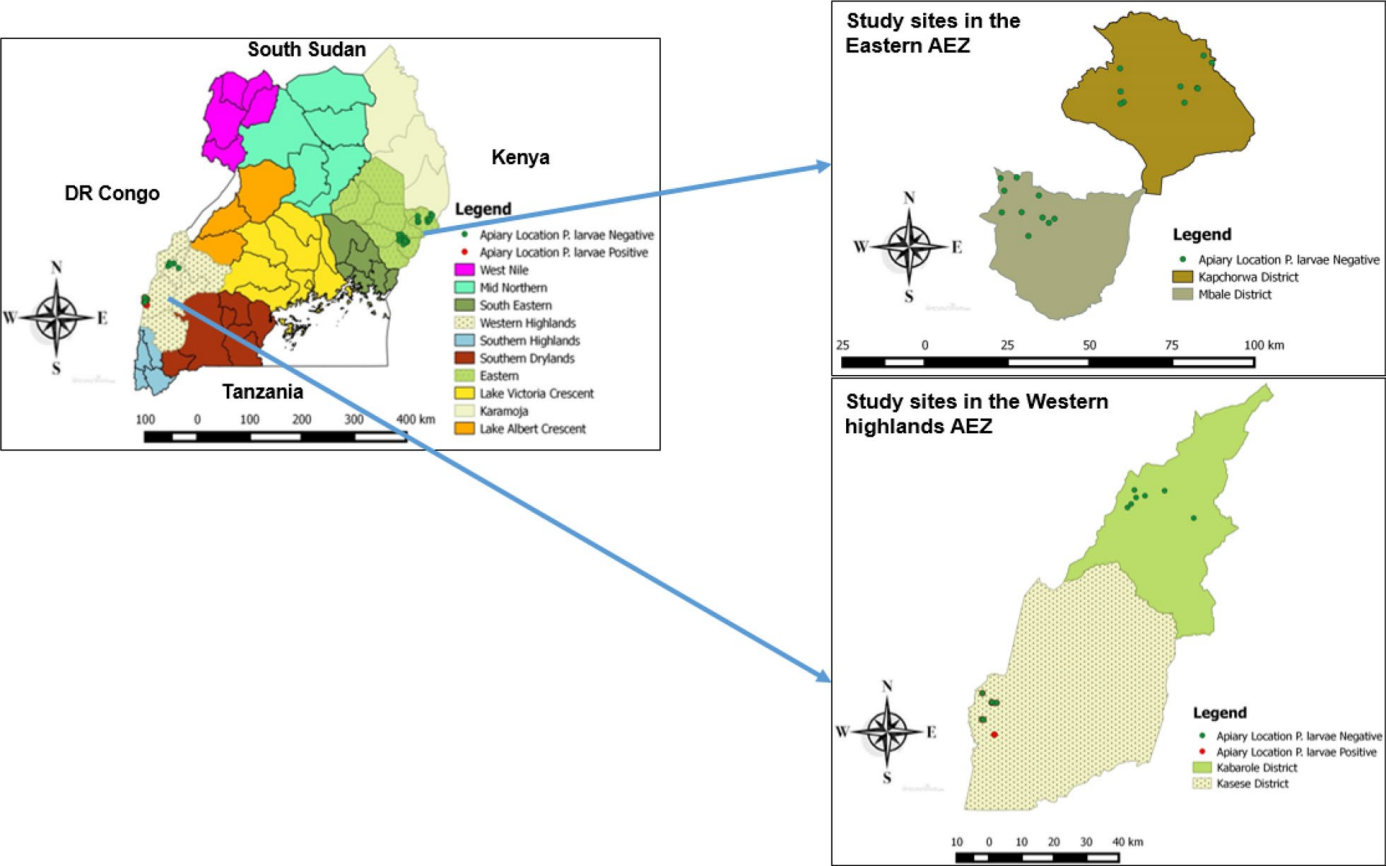
Location of study sites in the agro-ecological zones of Uganda and distribution of *P. larvae*

The study was conducted in the eastern and western highland agro-ecological zones (AEZ) of Uganda which are approximately 500 km apart (Fig. 1) but have comparable elevation, climatic conditions and land use activities. Altitude in the eastern agro-ecological zone ranges from 1000 to 4000 m above sea level (NEMA 2009) while in the western AEZ, it ranges from 600 to 4500 m (Chemurot et al. 2016). Both AEZs receive bimodal rainfall (900–2100 mm per year in the eastern and 875–1875 mm per year in the western AEZs) (Kajobe et al. 2009; Chemurot et al. 2016). Furthermore, in both AEZs beekeeping activities are practiced in farmlands and protected areas providing conditions for studying the possible influence of human activities on the prevalence of honeybee diseases. For this study, a farmland refers to public or private land under cultivation while protected areas are public owned lands that currently receive government protection because of their recognized natural and ecological values.

In each AEZ, two districts were selected based on having altitudinal gradients and varying land uses in beekeeping areas (Kasese and Kabarole in the western AEZ and Mbale and Kapchorwa in the eastern AEZ). At each district, sub-counties known for beekeeping activities were chosen in consultation with beekeeping extension workers. Then lists of beekeepers were obtained from the District Production Offices and the apiaries sampled were selected based on altitude and land uses. The altitude in the study apiaries ranged from 930–2400 m above sea level. Based on this, we stratified apiaries in each study district according to altitude into four strata; low (900–1100 m), mid-low (1101–1300 m), high (1301–1500 m) and very high (above 1500 m) and at least one apiary was sampled in each strata.

Two honeybee races, *Apis mellifera scutellata*, and *Apis mellifera adonsonii* were recently confirmed in Uganda and both races were identified in the eastern and western highland AEZs (Kasangaki et al. in Prep). We sampled a total of 67 honeybee colonies (56 top bar hives, 3 Langstroth and 8 fixed comb hives) from 33 apiaries during two sampling moments (dry and wet seasons) between December 2014 and September 2015 (Table 1). Only three honeybee colonies were sampled twice (during both seasons) because some colonies had either absconded or did not have brood at the time of second sampling. One brood comb from each honeybee colony was collected and frozen as soon as it was possible and later analyzed for *P. larvae* in the research laboratory at Ghent University, Belgium. During field work, observations on honeybee colony strength and productivity and clinical symptoms of AFB were recorded. Also, eight samples of honey imported and retailed in Uganda were collected directly from supermarkets in Mbale and Kabarole districts.

**Table 1:** Summary of the number of samples analysed for *P. larvae* during the two seasons and results obtained

S/N	Source of samples	Dry season		Wet season	
		Brood	Honey	Brood	Honey
1	Eastern AEZ	23 (0)	–	10 (0)	2 (0)
2	Western highland AEZ	36 (1)	–	1 (0)	3 (0)
3	Imported to Uganda	–	–	–	8 (0)

Figures indicate the number of samples analysed; those between brackets indicate the *P. larvae* positive samples found

### Culturing *P. larvae*

In the laboratory, the culture of *P. larvae* was performed according to routine protocols (de Graaf et al. 2006). Each brood sample was swabbed using cotton wool swabs (n = 10 cells; 5 on either side of the comb using two swabs) and the cotton wool washed in 5 ml Phosphate Buffered Saline (PBS). The sample was then heated in a water bath for 15 min at 80 °C and 150 µl pipetted onto MYPGP agar containing nalixidic (10 µg/ml) and pipemidic (20 µg/ml) acids. The agar was left to dry before being incubated at 37 °C for 4 days. All agar plates were observed for bacterial growth. Bacterial colonies were observed for similarities with *P. larvae* reference strain (LMG 9820). Suspicious colonies were subjected to catalase tests and those which were catalase negative were gram stained and examined at 1000× magnification on a microscope. Colonies were confirmed as *P. larvae* by PCR (Dobbelaere et al. 2001).

### PCR for *P. larvae*

A colony of the suspected bacterial sample was suspended in 50 µl of distilled water and heated to 100 °C for 10 min. The sample was then centrifuged at 13,300 rpm for 5 min and 1 µl of the supernatant was amplified in a 25 µl PCR mixture containing the following: 10× PCR buffer, 2.5 mM MgCl_2_, 50 pmol of each primer (AFB-F: 5′-CTTGTGTTTCTTTCGGGAGACGCCA-3′ and AFB-R: 5′-TCTTAGAGTGCCCACCTCTGCG-3′) (Dobbelaere et al. 2001), 400 pmol of each deoxynucleoside triphosphate, and 1.25 U of *Taq* polymerase. The PCR conditions consisted of a 94 °C (5 min) step; 30 cycles of 93 °C (1 min), 55 °C (1/2 min), and 72 °C (1 min); and a final cycle of 72 °C (10 min). As a positive control *P. larvae* LMG 9820 was used. The molecular weights of the PCR products were compared with those of the Generuler 1 kb plus marker on a 1 % agarose gel stained with ethidium bromide and visualized under ultraviolet light.

### ERIC genotyping

We performed ERIC genotyping following the procedures described by Genersch et al. (2006). Briefly, the DNA sequences of the primers used for *P. larvae* DNA fingerprinting were 5′-ATGTAAGCTCCTGGGGATTCAC-3′ (ERIC1R) and 5′-AAGTAAGTGACTGGGGTGAGCG-3′ (ERIC2). The PCR were carried out in final volumes of 25 µl consisting of 1× reaction buffer (Qiagen) and final concentrations of 2.5 mM MgCl_2_, 250 mM dNTPs, 10 mM primer and 0.3 U HotStarTaq polymerase (Qiagen). The reaction conditions were: an initial activation step (95 °C for 15 min); 35 cycles at 94 °C for 1 min, 53 °C for 1 min and 72 °C for 2.5 min, followed by a final elongation step at 72 °C for 10 min. A 10 µl sample from the PCR was analysed on a 0.8 % agarose gel. A positive control for each ERIC genotype was used (LMG 9820, R 20833, LMG 16252 and LMG 16247).

### Infection assay

The virulence test was conducted at the Laboratory of Molecular Entomology and Bee Pathology (L-MEB), Ghent University following the protocol described by de Graaf et al. (2013) using *Apis mellifera carnica* larvae. Briefly, plates each consisting of 24-wells were incubated at 34 °C for 24 h. A group of 30 larvae (in 3 wells) was treated with the Ugandan *P. larvae* isolate, another group of 30 larvae was treated with the *P. larvae* strain BRL 230010. Six (6) wells were left empty and filled with 1 ml of distilled water to avoid desiccation. Three hundred (300) µl of the spore-contaminated larval diet (20 spores of *P. larvae*/µl feed) was pipetted into each well of the treatment group. Three wells for the negative control group were left and fed on non-spore contaminated larval diet during the entire experiment. After 24 h of infection, larvae were transferred to a pre-warmed, fresh normal larval diet plate. The grafting tool was decontaminated between each group to avoid reinfection. Every treatment group received fresh larval diet every 24 h and the plates were analyzed each day under a stereo microscope to determine the health status of the larvae. Old feed was removed daily and replaced with pre-warmed fresh larval diet. After defecation (day 8), the larvae were transferred to pupation plates. Larvae were classified as dead when they stopped breathing (movement of tracheal openings stops) and lost body elasticity. The number of dead larvae was recorded every day. To determine whether *P. larvae* infection caused the death of a larva, dead larvae were plated out on MYPGP plates. Plates were incubated for 3 days at 37 °C to allow the growth of vegetative bacteria. Positive AFB infection was confirmed by growth of *P. larvae*. Further confirmation was provided by performing *P. larvae*-specific PCR-analysis of colonies grown from larval remains.

## Results

### Prevalence of *P. larvae*

**Fig. 2:**
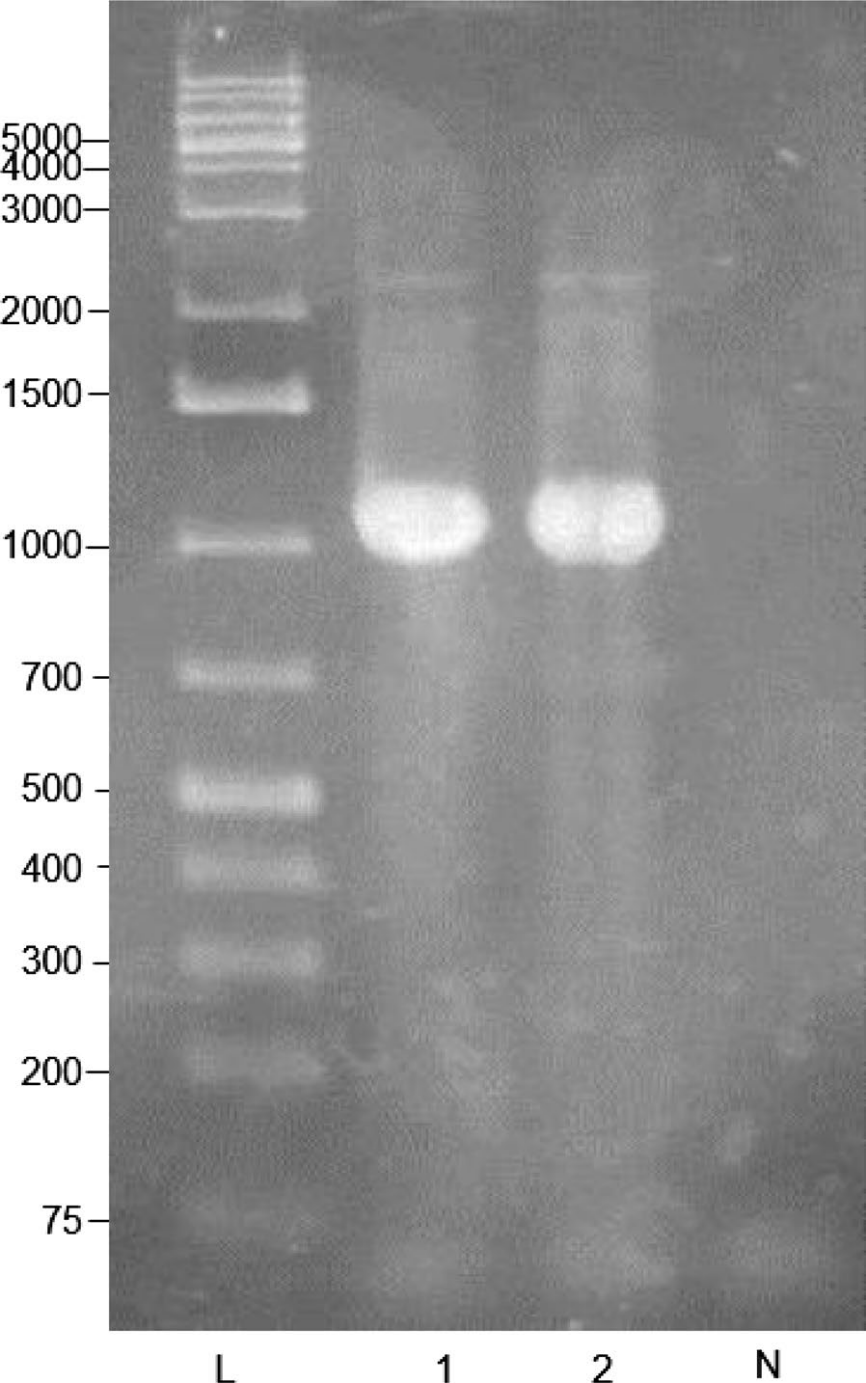
PCR product image: L = Generuler 1 kb plus marker, 1 = sample KAS-07, 2 = positive control (LMG 9820), N = negative control

The presence of *P. larvae* in samples of honeybee brood and honey from the two agro-ecological zones of Uganda is shown in Table 1. A total of 59 brood samples from the two agro-ecological zones were analyzed during the dry season. During the wet season, 11 brood and 13 honey samples were analyzed. No brood sample showed any clinical signs of AFB in the field. None of the honey samples were found to be contaminated with *P. larvae* spores (Table 1). Of the 59 honeybee brood samples analyzed during the dry season, only one (sample KAS-07) (representing 1.7 %) was confirmed positive for *P. larvae* (Table 1). As expected, the PCR product banded just above 1000-bp (around 1106-bp) on the Generuler 1 kb plus marker (Fig. 2).

### ERIC PCR and virulence assay

**Fig. 3:**
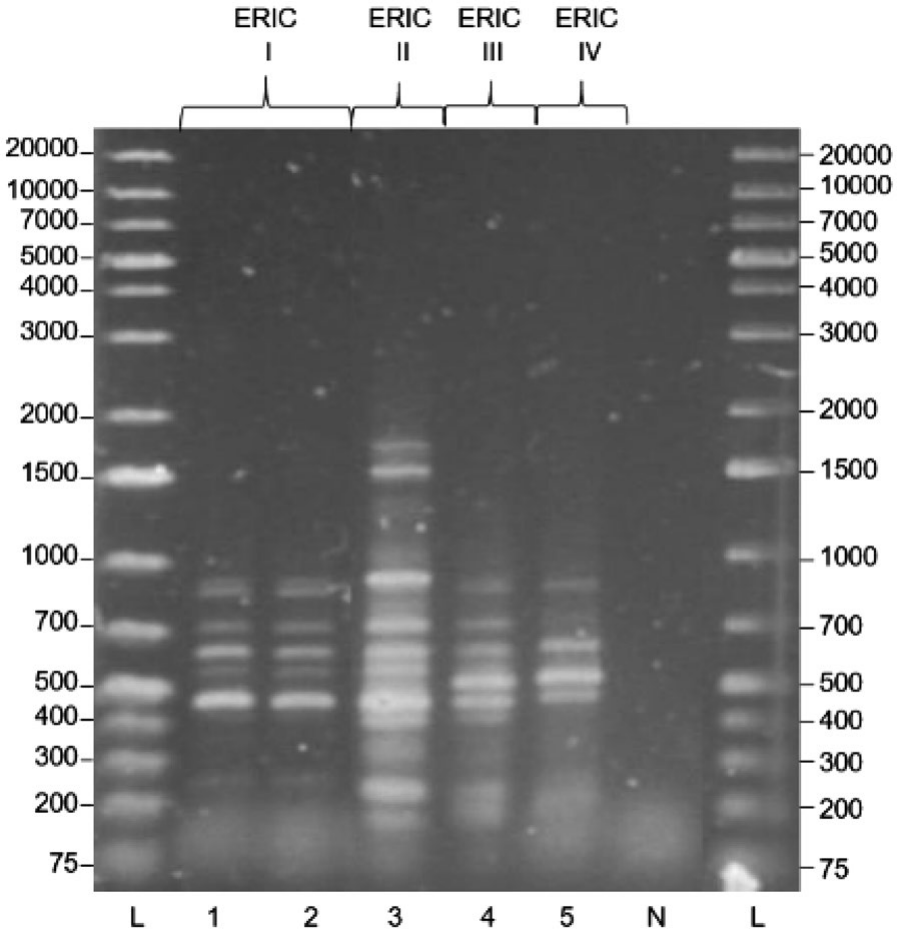
ERIC PCR product image: L = Generuler 1 kb plus marker, 1 = sample KAS-07, 2 = LMG 9820, 3 = R 20833, 4 = LMG 16252, 5 = LMG 16247, N = negative control

**Fig. 4:**
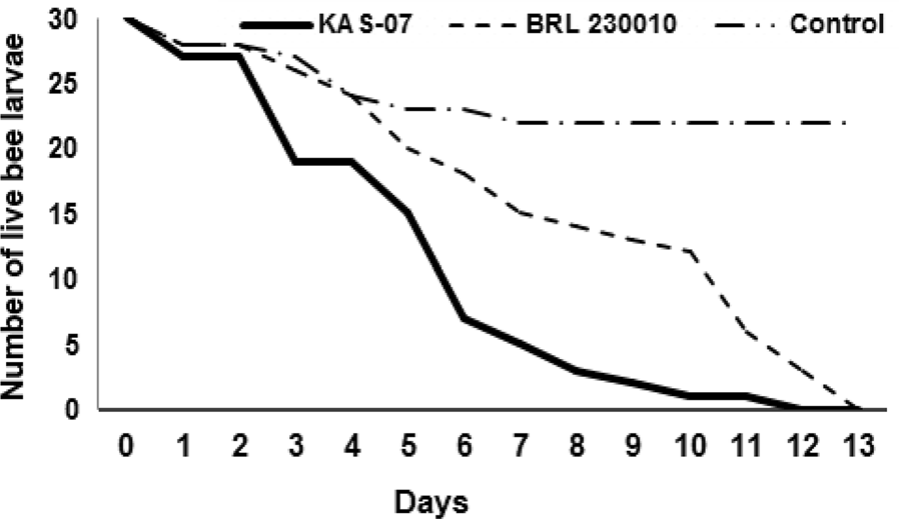
Comparison of the survival of *A. mellifera carnica* larvae feed on **KAS 07** Ugandan *P. larvae* strain found and ERIC I strain (BRL 230010)

Genotyping of the *P. larvae* strain found in this study revealed that it was an ERIC I strain (Fig. 3). The virulence of this *P. larvae* strain on *A. mellifera carnica* is shown in Fig. 2. Of the 30 honeybee larvae that were fed with spores, 25 had died by the 6th day (Fig. 4). For the reference ERIC I strain (BRL 230010), only 18 honeybee larvae had died in the same period. However, it is clear that by the 12th day, all honeybee larvae that were fed with *P. larvae* spores had died. This virulence assay finding confirms that the strain obtained is at least equally virulent when compared to BRL 230010, which was isolated from diseased colonies in the USA and which belongs to the ERIC I genotype (Qin et al. 2006).

## Discussion

In this study, 1.7 % of the honeybee colonies sampled during the dry season were positive for *P. larvae*. Being the first finding of *P. larvae* in Uganda, it was necessary to perform ERIC genotyping and virulence tests to compare this strain with reference strains found in some western countries. Moreover, as clinical signs were not observed, we wondered whether this strain had any disease causing potential. We also decided to conduct virulence tests of the *P. larvae* strain found on *A. mellifera carnica* of the research laboratory of Ghent University, Belgium as we did not have the facilities nor the required biosafety certificate to perform the experiments in Uganda. The results showed that the strain found was an ERIC I with at least equally high virulence when compared to BRL 230010 from the USA (Qin et al. 2006).

The prevalence (1.7 %) of *P. larvae* in honeybee colonies and (3.03 %) in apiaries recorded in this study is comparatively much lower than that reported in some Asian countries e.g. 37.3 % in honeybee colonies in Pakistan (Anjum et al. 2015), 24.8 % in honey samples from Taiwan (Chen et al. 2008) and some European countries e.g. 11 % in Belgium (de Graaf et al. 2001), 66 % in France (Mouret et al. 2013) and 5.3–9.8 % in Latvian apiaries (Chauzat et al. 2014; Laurent et al. 2015). However, the prevalence recorded in apiaries in our study sites is in the range of 1–5.7 % in Estonia, 1.5–4.5 % in Greece, 1.6–4.7 % in Poland, 2 % in Sweden, 2.6 % in Slovakia, 2.2–2.7 % in Italy (Chauzat et al. 2014; Laurent et al. 2015) and 1.6–3.2 % in Spain (Garrido-Bailón et al. 2013). Long term epidemiological studies show that the prevalence levels of AFB vary over time. For example, in Uruguay, AFB prevalence levels fluctuated over 12 years after it was first reported (Antúnez et al. 2012) suggesting that the levels recorded in Ugandan apiaries could change over time. Therefore, monitoring programs for this honeybee disease in Uganda should be developed and implemented to ensure that it is detected early and managed.

In Africa, AFB has been confirmed in South Africa (Human et al. 2011), Guinea Bissau (Hussein 2001; Hansen et al. 2003) and Egypt (Masry et al. 2014). *P. larvae* has also been detected in honey originating from Tunisia (Matheson 1993; Hussein 2001; Fries and Raina 2003; Hamdi et al. 2013), Algeria, Libya and Morocco (Hussein 2001). Despite *P. larvae* and other honeybee parasites like *Varroa destructor* being reported in Africa (Human et al. 2011; Muli et al. 2014; Strauss et al. 2015; Chemurot et al. 2016), no major colony losses have been reported yet. This could be associated with the higher levels of disease resistance in African honeybees (Human et al. 2011). However, such a trait may not persist if pathogens accumulate in hives especially with the promotion of frame beehives. Therefore, efforts should be made to prevent loss of the disease resistance in African honeybees.

Behavioral adaptations such as abscondment and swarming among African honeybee races may also explain their low levels of parasite infestation (Chemurot et al. 2016). Two honeybee races, *Apis mellifera scutellata*, and *Apis mellifera adonsonii* have been confirmed in Uganda (Kasangaki et al. in Prep). These honeybee races abscond from beehives more frequently when disturbed than other races of *A. mellifera* (Hansen and Brodsgaard 1997; reviewed in Dietemann et al. 2009). This behavioral trait could result in disinfection in honeybee colonies formally infected by *P. larvae* (Hansen and Brodsgaard 1997).

The higher levels of hygienic behavior of African honeybees may also reduce the level of AFB infection (Fries and Raina 2003; Human et al. 2011). In addition, the wax moth, *Galleria mellonella* which is a very common pest in Africa and only affects weak colonies (Strauss et al. 2013) may reduce AFB infestation levels by destroying large amounts of infected combs after colony abscondment (Hansen and Brodsgaard 1997; Human et al. 2011). The overall implication of this is that relatively very low AFB infection levels and extremely rare development of clinical symptoms are observed.

The *P. larvae* positive sample in this study was from a colony in a protected area suggesting that this pathogen could be present in feral honeybee colonies. Since beekeepers in Uganda rely on natural honeybee colonies to colonize their beehives (Chemurot 2011), *P. larvae* could spread from feral to managed colonies. On the other hand, absconding and swarming which are common among African honeybee races (Hansen and Brodsgaard 1997) could also spread this pathogen from managed to feral colonies. However, the current predominant use of traditional and top-bar beehives (Chemurot 2011) reduces chances of this pathogen accumulating in honeybee combs since beekeepers harvest the entire comb.

## Conclusion

Although honeybee health is an important theme in apiculture, only few attempts have been made to investigate honeybee diseases in Africa. This paper provides the first reported evidence in East Africa of AFB, one of the most serious honeybee diseases. We demonstrate that the pathogen detected is ERIC I strain of *P. larvae* and compare its virulence with a reference strain. The results suggest that the strain obtained was equally virulent on carniolan honeybees. We recommend regular countrywide monitoring and surveillance for *P. larvae* to ensure that this pathogen is detected in time and interventions made before it can cause major production losses to beekeepers.

## Abbreviations

AEZ: Agro-ecological zone
AFB: American foulbrood
DNA: Deoxyribonucleic acid
ERIC: Enterobacterial Repetitive Intergenic Consensus
PCR: Polymerase chain reaction

## References

[CR1] Adjlane N, Haddad N, Kechih S (2014). Comparative study between techniques for the diagnosis of American foulbrood (*Paenibacillus larvae*) in honeybee colony. J Anim Vet Adv.

[CR2] Anjum SI, Shah AH, Azim MK, Yousuf MJ, Khan S, Khan SN (2015). Prevalence of American foul brood disease of honeybee in north-west Pakistan. Biotechnol Biotec Eq.

[CR3] Antúnez K, Anido M, Branchiccela B, Harriet J, Campáb J, Zunino P (2012). American Foulbrood in Uruguay: twelve years from its first report. J Invertebr Pathol.

[CR4] Chauzat M, Laurent M, Rivière M, Saugeon C, Hendrikx P, Ribiere-Chabert M (2014). Epilobee A pan-European epidemiological study on honeybee colony losses 2012–2013. Commission Européene.

[CR5] Chemurot M (2011). Beekeeping in Adjumani district, Uganda. Bee World.

[CR6] Chemurot M, Akol AM, Masembe C, De Smet L, Descamps T, de Graaf DC (2016). Factors influencing the prevalence and infestation levels of *Varroa destructor* in honeybee colonies in two highland agro-ecological zones of Uganda. Exp Appl Acarol.

[CR7] Chen Y, Cheng H, Huang C (2008). American foulbrood spores in honey samples in Taiwan. Formos Entomol.

[CR8] de Graaf DC, Vandekerchove D, Dobbelaere W, Peeters JE, Jacobs F (2001). Influence of the proximity of American foulbrood cases and apicultural management on the prevalence of *Paenibacillus larvae* spores in Belgian honey. Apidologie.

[CR9] de Graaf DC, Alippi AM, Brown M, Evans JD, Feldlaufer M, Gregorc A (2006). Diagnosis of American foulbrood in honey bees: a synthesis and proposed analytical protocols. Lett Appl Microbiol.

[CR10] de Graaf DC, Alippi AM, Antúnez K, Aronstein KA, Budge G, De Koker D (2013). Standard methods for American foulbrood research. J Apicult Res.

[CR11] Dietemann V, Pirk CWW, Crewe R (2009). Is there a need for conservation of honeybees in Africa?. Apidologie.

[CR12] Dobbelaere W, de Graaf DC, Peeters J (2001). Development of a fast and reliable diagnostic method for American foulbrood disease (*Paenibacillus larvae* subsp. *larvae*) using a 16S rRNA gene based PCR. Apidologie.

[CR13] Ellis JD, Munn PA (2005). The worldwide health status of honey bees. Bee World.

[CR14] Fries I, Raina S (2003). American foulbrood and African honey bees (Hymenoptera: Apidae). J Econ Entomol.

[CR15] Fries I, Lindström A, Korpela S (2006). Vertical transmission of American foulbrood (*Paenibacillus larvae*) in honey bees (*Apis mellifera*). Vet Microbiol.

[CR16] Garrido-Bailón E, Higes M, Martínez-Salvador A, Antúnez K, Botías C, Meana A (2013). The prevalence of the honeybee brood pathogens *Ascosphaera apis*, *Paenibacillus larvae* and *Melissococcus plutonius* in Spanish apiaries determined with a new multiplex PCR assay. Microbiol Biotechnol.

[CR17] Genersch E (2010). American Foulbrood in honeybees and its causative agent, *Paenibacillus larvae*. J Invertebr Pathol.

[CR18] Genersch E (2010). Honey bee pathology: current threats to honey bees and beekeeping. Appl Microbiol Biot.

[CR19] Genersch E, Ashiralieva A, Fries I (2005). Strain and genotype-specific differences in virulence of *Paenibacillus larvae* subsp. *larvae*, a bacterial pathogen causing American foulbrood disease in honeybees. Appl Environ Microbiol.

[CR20] Genersch E, Forsgren E, Pentikäinen J, Ashiralieva A, Rauch S, Kilwinski J, Fries I (2006). Reclassification of *Paenibacillus larvae* subsp. *pulvifaciens* and *Paenibacillus larvae* subsp. *larvae* as *Paenibacillus larvae* without subspecies differentiation. Int J Syst Evol Microbiol.

[CR21] Hamdi C, Essanaa J, Sansonno L, Crotti E, Abdi K, Barbouche N, Balloi A, Gonella E, Alma A, Daffonchio D, Boudabous A, Cherif A (2013) Genetic and biochemical diversity of *Paenibacillus larvae* Isolated from tunisian infected honey bee broods. BioMed Res Int vol. 2013, Article ID 479893, 9 pages, 2013. doi:10.1155/2013/479893 10.1155/2013/479893 PMC3774041 24073406

[CR22] Hansen H, Brodsgaard C (1997). The spread and control of American foulbrood. Bees Dev J.

[CR23] Hansen H, Brodsgaard CJ, Kryger P, Nicolaisen M (2003). A scientific note on the presence of *Paenibacillus larvae larvae* spores in sub-Saharan African honey. Apidologie.

[CR24] Human H, Pirk CWW, Crewe RM, Dietemann V (2011). The honeybee disease American foulbrood—an African perspective. Afr Entomol.

[CR25] Hussein MH (2001) Beekeeping in Africa: i-north, east, north-east and west African countries. In: Proceedings of 37th international apicultural congress, 28 Oct–1 Nov 2001, Durban, South Africa

[CR26] Jacobs F, Simoens C, de Graaf DC, Deckers J (2006). Scope for non-wood forest products income generation from rehabilitation areas: focus on beekeeping. J Drylands.

[CR27] Kajobe R, Agea JG, Kugonza DR, Alioni V, Otim SA, Rureba T (2009). National beekeeping calendar, honeybee pest and disease control methods for improved production of honey and other hive products in Uganda.

[CR28] Laurent M, Hendrikx P, Ribiere-Chabert M, Chauzat M (2015). A pan-European epidemiological study on honeybee colony losses 2012–2014.

[CR29] Lindstrom A, Korpela S, Fries I (2008). Horizontal transmission of *Paenibacillus larvae* spores between honey bee (*Apis mellifera*) colonies through robbing. Apidologie.

[CR30] Masry SHD, Kabeil SS, Hafez EE (2014). New *Paenibacillus larvae* bacterial isolates from honey bee colonies infected with American foulbrood disease in Egypt. Biotechnol Biotec Eq.

[CR31] Matheson A (1993). World bee health report. Bee world.

[CR32] Matheson A (1996). World bee health update. Bee World.

[CR33] Morrissey BJ, Helgason T, Poppinga L, Fünfhaus A, Genersch E, Budge GE (2015). Biogeography of *Paenibacillus larvae*, the causative agent of American foulbrood, using a new multilocus sequence typing scheme. Environ Microbiol.

[CR34] Mouret C, Lambert O, Piroux M, Beaudeau F, Provost B, Benet P (2013). Prevalence of 12 infectious agents in field colonies of 18 apiaries in western France. Revue Méd Vét.

[CR35] Muli E, Patch H, Frazier M, Frazier J, Torto B, Baumgarten T (2014). Evaluation of the distribution and impacts of parasites, pathogens, and pesticides on honey bee (*Apis mellifera*) populations in east Africa. PLoS ONE.

[CR36] NEMA (2009) Uganda atlas of our changing environment. Kampala: National Environment Management Authority (NEMA) NEMA House, Jinja Road P.O. Box 22255 Kampala Uganda

[CR37] Pirk CWW, Strauss U, Yusuf AA, Demares F (2015). Honeybee health in Africa—a review. Apidologie.

[CR38] Qin X, Evans JD, Aronstein KA, Murray KD, Weinstock GM (2006). Genome sequences of the honey bee pathogens *Paenibacillus larvae* and *Ascosphaera apis*. Insect Mol Biol.

[CR39] Strauss U, Human H, Gauthier L, Crewe RM, Dietemann V, Pirk CWW (2013). Seasonal prevalence of pathogens and parasites in the savannah honeybee (*Apis mellifera scutellata*). J Invertebr Pathol.

[CR40] Strauss U, Pirk CWW, Crewe RM, Human H, Dietemann V (2015). Impact of *Varroa destructor* on honeybee (*Apis mellifera scutellata*) colony development in South Africa. Exp Appl Acarol.

[CR41] UEPB (2005) Uganda Apiculture Export Strategy. (U. E. P. Board, Ed.)Uganda Apiculture Export Strategy, Kampala

[CR42] Woodrow AW (1942). Susceptibility of honeybee larvae to individual inoculations with spores of *Bacillus larvae*. J Econ Entomol.

